# Systematic review: The management of unhealed wounds and persistent perineal sinuses following proctectomy in inflammatory bowel disease

**DOI:** 10.1007/s10151-025-03242-z

**Published:** 2025-12-08

**Authors:** T. Pelly, E. Anand, S. Holubar, P. Tozer, A. Hart

**Affiliations:** 1 Robin Phillips Fistula Research Unit, St Mark’s the National Bowel Hospital, London, UK; 2 Imperial College London, London, UK; 3 Cleveland Clinic, Cleveland, OH USA

**Keywords:** Fistulizing perianal Crohn’s disease, Perianal abscess

## Abstract

**Introduction:**

Unhealed wounds and persistent perineal sinuses (PPS) may occur in as many as one third of patients after proctectomy for Crohn’s disease. The management of these conditions remains a significant challenge, particularly in the context of inflammatory bowel disease (IBD), with existing therapies plagued by high failure rates. This systematic review of the literature assessed the efficacy of medical and surgical therapy for PPS closure in IBD. Secondary aims included review of classification systems used for PPS.

**Methods:**

A literature search was conducted using Medline, Embase and Cochrane databases on 17 December 2024. The review was registered on PROSPERO (CRD42024622582). Inclusion criteria were adult patients with IBD and PPS or unhealed wounds following proctectomy. We excluded abstract-only publications, case reports, cancer and paediatric cohorts. Two reviewers independently screened abstracts and full texts and extracted data. The primary outcome was clinical healing rate. Secondary outcomes included classification systems used to describe PPS. Risk of bias was assessed.

**Results:**

Of 496 records identified, following removal of duplicates, 489 abstracts were screened, and 60 full text articles assessed for eligibility. Of 25 articles included in the final analysis, 23 were case series or retrospective cohort studies, and all were at high risk of bias. No randomised controlled trials were identified. Five articles (including two of the case series) described classification systems for PPS. Interventions included hyperbaric oxygen therapy, Karydakis flap, cleft closure, omentoplasty, skin grafting, gracilis and rectus abdominis flap, platelet-derived growth factor, curettage, lay open and excision of sinuses. Reported healing rates ranged from 30% to 100%. Heterogeneity in the reporting of outcomes, as well as the interventions performed precluded meta-analysis.

**Conclusion:**

The published evidence for treatment of PPS in IBD consists of low-quality evidence case series with high risk of bias. There is a need for standardised outcome reporting and high-quality, prospective studies to establish effective treatment algorithms.

## Introduction

Since 1908, when Ernest Miles first described the abdominoperineal excision of the rectum, unhealed perineal wounds and persistent perineal sinuses (PPS) have been recognised as challenging complications, particularly in the context of inflammatory bowel disease (IBD) [1, 2]. Following proctectomy for perianal Crohn’s disease (pCD), between one quarter and one third of patients’ wounds may not heal by 12 months [3, 4]. Symptoms from unhealed wounds and PPS have a significant impact on quality of life, and include ongoing discharge, pain and bleeding [5].

Tolstedt recognised PPS as a “disability serious enough to cause repeated hospitalization for surgical treatment” and wounds have traditionally been regarded as delayed or non-healed if they have not healed by 6 months [6]. This chronological definition was originally described by Watts, Dombal and Goligher in 1966, and the authors commented that this was “arbitrary” at the time of publication [7]. More recently, in the context of pCD, the TOpClass consortium classified persistent symptoms following proctectomy as Class 4 pCD, which is further subdivided into Class 4a—suitable for repair, and Class 4b—suitable for symptom control [8]. Unhealed wounds and PPS following surgery are varied and complicated anatomically, and may involve a number of other organs, and involvement of the sacrum often results in osteomyelitis [5]. Accurate classification is necessary to guide clinicians in selecting the most appropriate treatment options. Terminology relating to PPS is varied in the literature, confusingly, with definitions of sinuses and delayed, poorly healing or non-healing wounds often used interchangeably. For the purpose of this review, we refer to “unhealed wound/PPS” throughout to encompass the complete spectrum of the pathology that may be encountered.

Several treatments have been described to treat unhealed wounds/PPS after proctectomy for IBD. These include advanced medical therapies, curettage, excision of the sinus, skin grafting, reconstruction with a myocutaneous flap, as well as novel treatments such as hyperbaric oxygen therapy (HBOT) [5]. There are no current guidelines to aid clinicians in selecting treatments. In order to plan effective future research into treating these challenging conditions, there is a need systematically to review and appraise the evidence for the available treatments.

### Objectives

The primary aim of this systematic review was to assess the literature for the effectiveness and outcomes of various medical, surgical, and alternative treatment modalities for managing unhealed wounds/PPS following proctectomy for IBD. The secondary aim of this review was to identify classification systems for these conditions following proctectomy for IBD.

## Methods

The review was registered on PROSPERO (CRD42024622582). A literature search strategy was developed and carried out according to PRISMA guidance, and in collaboration with an information specialist from the Royal College of Surgeons.

### Eligibility criteria

Inclusion criteria were studies involving adults (> 18 years) with either Crohn’s disease (CD) or ulcerative colitis (UC) who had undergone proctectomy, pan-proctocolectomy, completion proctectomy or ileoanal pouch excision and subsequently developed unhealed wounds/PPS, with subsequent treatment. Exclusion criteria were studies where patients had surgery for indications other than IBD, e.g. cancer, and this was not reported separately. Studies describing interventions such as myocutaneous flaps at the time of proctectomy were excluded. Case studies were excluded.

### Information sources

Information sources were MEDLINE, EMBASE, CENTRAL, CDSR and Trip Pro databases. These sources were searched on 17 December 24.

### Search strategy

An example search strategy is presented in Table 1.

**Table 1:** Search strategy (MEDLINE)

1	Proctocolectomy, Restorative/
2	(Proctectom* or Post-proctectom* or Postproctectom* or Proctocolectom* or Panproctocolectom* or Pan-proctocolectom*).ti,ab,kw,kf
3	1 or 2
4	exp Inflammatory Bowel Diseases/
5	(inflammatory bowel disease* or IBD or Crohns or crohn’s or ulcerative colitis or UC).ti,ab,kw,kf
6	4 or 5
7	3 and 6
8	(Perineum/ or Rectum/) and (Postoperative Complications/ or Wound Healing/ or Wound Closure Techniques/)
9	(perineal sinus* or PPS or perineal wound*).ti,ab,kw,kf
10	8 or 9
11	3 and 6 and 10

### Selection process

A minimum of two reviewers independently screened each title, abstract and full text article using the Covidence software for systematic reviews [9].

### Data collection process and data items

Two reviewers independently extracted data from each included article using a pre-specified template on the Covidence software. Extracted data included study population size, whether the study population was patients with CD, UC, or both, intervention types, healing rates and duration of follow-up.

### Risk of bias

Risk of bias (ROB) was assessed for each article using the National Institutes of Health (NIH) quality assessment tool, which was deemed the most suitable assessment tool given the high numbers of retrospective case series [10]. Certainty assessment was assessed using the GRADE framework. Articles that were included as they described a classification system were not assessed for risk of bias.

### Synthesis methods

**Fig. 1:**
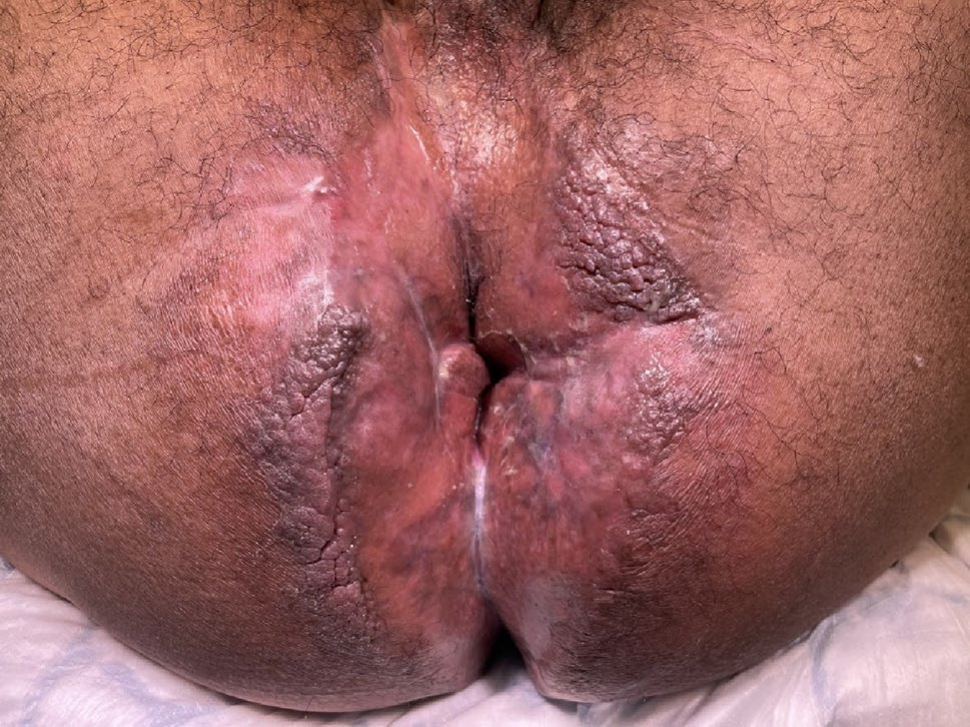
Persistent perineal sinus following proctectomy for perianal Crohn’s disease

No synthesis or meta-analysis was performed because of the general low quality and heterogeneity of the included studies, namely differing therapies, timepoints and definitions of outcomes such as clinical healing (Fig. 1).

## Results

### Study selection

**Fig. 2:**
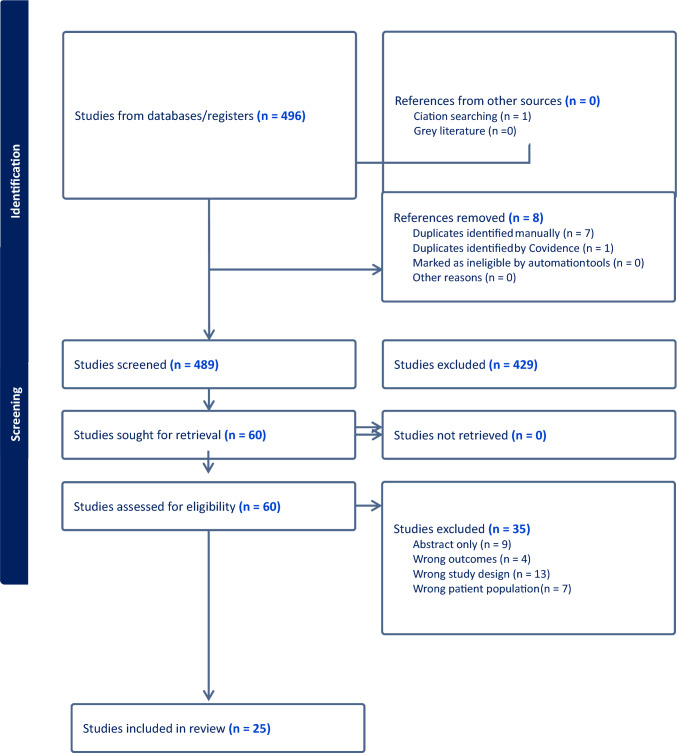
PRISMA flowchart

MEDLINE, Embase, CENTRAL, CDSR and Trip Pro databases were searched on 17 December 2024. This search yielded 496 records after the removal of 124 duplicates. These records were uploaded to Covidence software, where a further eight duplicates were removed. A total of 489 titles and abstracts were screened (Fig. 2, PRISMA chart).

Sixty eligible full text articles were included, and of these, 25 articles were included in the systematic review, with one of these articles identified by citation tracking. Twenty-three were case series or retrospective cohort studies, and two were literature reviews or expert consensus processes. Of all the studies, five included a classification of unhealed wounds/PPS. At title and abstract screening, inter-reviewer agreement was 0.93 (Cohen’s kappa 0.67), indicating substantial agreement. At full-text screening, inter-reviewer agreement was 0.93 (Cohen’s kappa 0.78) indicating substantial agreement.

### Study characteristics

There were 25 studies included for full-text review; 22 (95%) were retrospective studies, 1 (5%) was a prospective case series, 1 (4%) was an expert consensus process, and 1 (4%) was a systematic review. No randomised controlled trials were identified. The mean sample size was *N* = 11. The mean length of follow-up was 32 months (range 1–275).

### Risk of bias in studies

Of the 25 studies, 23 were clinical studies and therefore eligible for ROB assessment. In terms of quality of 23 of the included clinical studies, the majority 18/23 (78%) were regarded as ‘poor’ methodological quality on assessment with the NIH assessment tool; 4 (17%) were of ‘fair’ quality, and one was ‘good’.

### Certainty of evidence

A certainty assessment was performed for the intervention with the highest number of studies, those investigating musculocutaneous flaps. According to the GRADEpro GDT (GRADEpro Guideline Development Tool [11]), the certainty of evidence was assessed to be “very low”.

### Results of individual studies

Across the 23 clinical studies identified in the literature search, 22 studies investigated interventions for unhealed wounds/PPS including simple excision, laying open of tracts, curettage, radical excision, gracilis and rectus abdominis muscle flaps, skin grafting, HBOT, fibrin glue, platelet-derived growth factor (PDGF), omentoplasty, Karydakis flap, and modified cleft closure (Table 2). These procedures were sometimes performed in combination, and often outcomes were not reported separately. The range of healing rates for patients with IBD in this cohort was 15–100%, with the majority of studies reporting good outcomes. Any further data synthesis or meta-analysis was prohibited by heterogeneity in the data, including the definition of healing, timings and reporting of outcomes.

**Table 2:** Articles included in the systematic review

Study ID	Intervention	Design	Population	Terminology	Number (IBD only)	Follow-up period	Healing rate	NIH assessment	Medical therapy at time of intervention
Lansdorp 2020 [22]	HBOT (40 sessions, 2.4 atm)	Prospective case series	CD	Perineal metastatic CD	3	3 months	33%	Fair	All patients had prior biologic treatment. No patients on biologics at the time of treatment
Lubbers 1982 [31]	Curettage/laying open of tracts/excision of tracts	Retrospective case series	Mixed	Healed/unhealed/persistent sinus	11	Not clearly defined	64%	Poor	28.6 had steroid therapy at time of proctectomy. None reported at time of intervention for PPS
Scammell 1986 [32]	Multiple procedures (flap, local excision, fibrin glue)	Retrospective case series	CD	Delayed/unhealed wound/PPS	21	Not clearly defined	41.7%	Poor	30% had steroid at time of proctectomy. None reported at time of intervention for PPS
Kirkegaard 1983 [21]	Fibrin glue	Retrospective case series	Mixed	Perineal sinus	9	Average 3 months (0–5)	89%	Poor	Not reported
Ambrose 1988 [20]	Fibrin glue	Retrospective case series	Mixed	PPS	10	Average 22 months	30%	Poor	Not reported
Kurtz 2011[26]	Rh PDGF application	Retrospective case series	Mixed	Poor/delayed/unhealed wounds	10	Not clearly reported	60%, 100% after second procedure	Fair	Not reported
Yamamoto 1999 [33]	Multiple procedures (drainage, radical excision with coccygectomy, flaps, omentoplasty (classification)	Retrospective case series	CD	Early/delayed healing/PPS	33	157 months (1–275 months)	29% for radical excision and closure, 50% for RAM flap and 50% for omentoplasty	Poor	49% had steroid at time of proctectomy. None reported at time of intervention for PPS
Lohsiriwat 2008 [30]	Multiple interventions, 11 curettage, 6 VAC, 3 sinus excision, 3 lay open, 2 laparotomy, 1 gracilis flap	Retrospective case series	Mixed	PPS	14	At least 6 months	64%	Fair	31% had immunosuppressive therapy at time of pouch excision, 43% at time of intervention for PPS
Branagan 2006 [24]	Modified cleft closure	Retrospective case series	Mixed	PPS	5	Mean 43.9 months (range 6—79 months)	80% (one patient requiring repeat procedure	Poor	Not reported
Au 2016 [39]	Karydakis flap	Retrospective case series	CD	PPS	2	Average 12 months	100%	Poor	Not reported
Yamamoto 2001 [25]	Omentoplasty	Retrospective case series	CD	PPS	6	28 months (23–32)	83.3%	Poor	Not reported
Anderson 1976 [28]	Skin grafting	Retrospective case series	Mixed	Unhealed wound	48	Not clearly reported	91% “good”, of which 15% were “completely healed”	Poor	Corticotropin (ACTH) administered to all patients until discharge
Oakley 1985 [29]	Curettage/excision and skin grafting	Retrospective case series	UC	Unhealed wound/delayed healing	35	Not clearly defined	61%	Poor	Not reported
McLeod 1985 [27]	Split-thickness skin grafting	Retrospective case series	Mixed	Chronic perineal sinus	9	Average 55 months (5–12 years)	87.5%	Poor	Not reported
Bartholdson 1975 [17]	Gracilis muscle transposition	Retrospective case series	Mixed	PPS	4	3 months	50%	Poor	Not reported
Lens 1979 [15]	Gracilis muscle flap	Retrospective case series	Mixed	Delayed wound healing/unhealed wound/PPS	3	average 9 months	67%	Poor	Not reported
Baek 1981 [13]	Gracilis muscle flap	Retrospective case series	Mixed	Unhealed wound/PPS	5	8 months	100%	Poor	Not reported
Anthony 1990 [14]	Gracilis and gluteus maximus flap (classification)	Retrospective case series	Mixed	Delayed/nonhealing/chronic wounds	5	Average 3.5 years	100%	Poor	Not reported
Loessin 1995 [18]	Inferiorly based trans-pelvic rectus abdominis muscle or musculocutaneous flap	Retrospective case series	UC/CD ? mixed	Persistent perineal/sacral defects	4	Average 32 (6–56)	100% (75% “excellent, 25% “fair”)	Poor	80% had immunosuppressive treatment
Rius 2000 [16]	Gracilis muscle flap	Retrospective case series	CD	Unhealed wound/PPS	3	18 months	100%	Poor	66% of patients had previous steroid therapy
Maeda 2011 [40]	Gracilis muscle flap	Retrospective case series	CD	Persistent non-healing perianal sinus/non-healing perineum	4	Average 64 months (23–123)	75% (healed unhealed)	Fair	No patients on medical therapy
Chan 2014 [12]	RAM flap and perioperative HBOT	Retrospective case series	Mixed	PPS	4	Average 35 months (8–64)	100%	Fair	25% of patients had previous infliximab therapy

*ACTH* adrenocorticotropic hormone,* CD* Crohn’s disease,* HBOT* hyperbaric oxygen therapy,* IBD* inflammatory bowel disease,* PPS* persistent perineal sinus,* RAM* rectus abdominis muscle,* Rh PDGF* recombinant human platelet-derived growth factor,* UC* ulcerative colitis,* VAC* vacuum-assisted closure

#### Musculocutaneous flaps

Eight studies reported the use of musculocutaneous flaps, including gracilis muscle, rectus abdominis muscle (RAM), and gluteus muscle flaps in patients with unhealed wounds/PPS [12–19]. Six of the eight studies were poor in quality with a high risk of bias, and the population sizes overall were small, ranging from three to five patients. Five studies reported 100% success rates, but all were rated as poor quality using the NIH assessment. Maeda et al. performed a retrospective case series of patients with complex fistulae, unhealed wounds or PPS [19]. The four patients with unhealed wounds/PPS were well described, with follow-up ranging from 30 to 123 months. Structured telephone follow-ups were performed and 75% of patients had healing, with high satisfaction ratings. Chan et al. reported on a series of four patients with PPS who had preoperative HBOT followed by abdominoperineal PPS excision and perineal reconstruction with a RAM flap [12]. This was one of the few studies with an a priori definition of wound healing as complete epithelialization of the wound with cessation of discharge; for the remaining studies, healing was not defined, and often it was unclear whether this assessment was made in clinic or on retrospective review of documentation.

#### Fibrin glue

Fibrin glue was assessed in two of the case series. Ambrose et al. administered Tisseel® fibrin glue to 10 patients with PPS after proctectomy for UC or CD [20]. Three patients were found to have healed, three were improved, two were unchanged on follow-up. In two patients, enteroperineal fistulae were identified after the procedure that had not been identified preoperatively. Failure of the treatment was felt to be due to sinuses that were too large. Kirkegaard et al. report a series of nine patients treated with fibrin glue, with success in eight out of them [21]. In both series, failure of the treatment to improve symptoms was put down to the sinus or cavity being too large, suggesting that this treatment may be an effective option in patients with PPS not fit enough for other treatments, who have narrow and relatively straight sinuses.

#### Hyperbaric oxygen therapy

HBOT was assessed in one case series, reported by Lansdorp et al., in addition to the study by Chan et al. describing perioperative HBOT in RAM flaps. Lansdorp et al. recruited three patients with biopsy-proven CD of the perineal wound following proctectomy, who underwent 40 daily sessions of HBOT at 2.4 atm [22]. One patient had complete healing, another had a recurrence 3 months after treatment, and one patient had improvement only on a series of patient-reported outcome measure (PROM) questionnaires. The treatment was reported as well tolerated in all patients. Although there is evidence for the efficacy of HBOT for other indications, larger prospective studies should be performed before HBOT can be recommended for this indication, and consideration given to combination surgical repair and HBOT [23].

#### Cleft closure and Karydakis flap

Two studies reported on applications of techniques traditionally associated with pilonidal disease surgery to PPS. Branagan et al. describe the use of Bascom’s cleft closure technique in patients with PPS. In these patients, healing was achieved on the initial attempt in four patients, with one patient requiring a subsequent procedure [24]. Au et al. report use of the Karydakis flap involving an asymmetrical elliptical incision to excise the sinus followed by closure, in two patients with PPS, with both (100%) achieving healing [39].

#### Omentoplasty

Excision of PPS and abdominal omentoplasty was performed in a series of six patients by Yamamoto et al. [25]. At a median follow-up of 28 months, sinuses were healed in 5/6 (83%) patients. Although the series was small and quality of evidence poor using the NIH assessment, the procedure appeared to be effective. However, it should be noted that the abdominal approach required has inherent risks of iatrogenic visceral injury in the post-proctectomy abdomen.

#### Platelet-derived growth factor

One series reported by Kurtz et al. assessed the use of PDGF, with a 60% healing rate in 10 patients with unhealed wounds after proctectomy [26]. Similar to the application of fibrin glue, the authors suggest that this may be a suitable option for patients with smaller sinuses. It should be noted that this is contraindicated in patients with cancer so should not be used in cases of proctectomy or pan-proctocolectomy due to dysplasia.

#### Skin grafting

Two case series report the use of skin grafting to treat unhealed wounds/PPS complicating proctectomy for IBD. McCleod et al. report on a series of nine patients undergoing excision of the PPS [27]. Of these patients, seven achieved healing, with one patient improved but still “disabled by the persistent pelvic pain”. Timing of the grafting was inconsistent, with immediate grafting performed in some patients, and in others it was delayed. Four patients required further procedures including repeat grafting. Anderson et al. describe an approach where the wound was debrided and a skin graft applied over the “saucerized” portion of the wound, but not the sinus [28]. A good result was defined as epithelialization of the wound, with only the sinus remaining unhealed. The authors acknowledge that a positive finding may not relieve discharge completely in 80% of patients. In this relatively large series of 49 patients, 44 were judged to have had a good result, although 13 required repeat procedures. Oakley et al. report a 75% healing rate in a cohort of 12 patients with PPS following UC [29].

#### Multiple procedures

Several studies investigated multiple procedures performed in a cohort of patients with unhealed wounds/PPS. Lohsirwat et al. described a total of 26 procedures, including curettage, VAC devices, sinus excision, laying open, laparotomy and gracilis flap construction. A pooled healing rate was reported of 64%. Although they did not report results for individual treatments, they reported that the most successful treatment was simple curettage, although they often required multiple treatments [30]. Lubbers et al. reported on a series of 11 patients undergoing surgical procedures for PPS, including abscess drainage, excision, curettage, laying open of tracts, and two flaps and grafts [31]. Some of these patients required as many as four or five procedures for healing. Curettage was less successful in this series, failing on eight occasions. Scammell et al. report a series of 10 patients with unhealed wounds/PPS, who had a number of procedures including excision of sinuses, excision of the coccyx, myocutaneous flap, fibrin glue and skin grafting [32]. Results for individual treatments and the combinations in which these were used were not reported. Yamamoto et al. reported 56 procedures in 24 patient, including drainage, coccygectomy, flaps and omentoplasty [33]. Healing rates were similar across these groups, although it is interesting to note that longer sinuses (> 10 cm) were less frequently cured with surgical treatment.

### Classification

Five different classification systems were identified (Table 3). These include the TOpClass classification, the Watts-Goligher classification, temporal classifications described by Yamamoto et al. and Anthony et al., as well as an anatomical classification described by Wilson et al. [14, 33–35, 38].

**Table 3:** Classification systems for persistent perineal sinus

Study	Study type	Type of classification
Watts 1966 [38]	Retrospective case series	Temporal
Yamamoto 1999 [33]	Retrospective case series	Temporal
Anthony 1990 [14]	Case series	Temporal
Wilson et al. [35]	Systematic review	Anatomical
TOpClass classification [34]	Expert consensus	Hybrid disease characteristic/management/clinician and patient goals

## Discussion

### General interpretation of results in the context of evidence

This systematic review highlights the considerable variability in the management and reported outcomes of unhealed wounds and PPS after proctectomy in IBD. Across 23 studies, a wide range of surgical and adjunctive interventions were described, including musculocutaneous flaps, skin grafting, HBOT, cleft closure techniques, omentoplasty, PDGF, and fibrin glue. Musculocutaneous flaps were the most frequently studied intervention, with several small case series reporting healing rates of 50–100%, though all but two were assessed as poor quality on the basis of NIH criteria.

This review has also identified a number of classification systems relating to unhealed wounds/PPS. However, existing clinical classifications and definitions require further work to standardise them for use in future research.

### Limitations of included evidence

#### Treatment outcomes

Despite some studies suggesting promising outcomes, the overall quality of evidence remains low, with significant heterogeneity in study design, definitions of healing, follow-up durations, and outcome reporting. Given these inconsistencies, a meta-analysis would be misleading, preventing the derivation of pooled healing rates. Instead, these findings highlight the need for standardised definitions (including clarifying definitions of wound healing and sinuses, classification, interventions, PROMs, outcomes and their measurement), prospective studies, and higher-quality evidence to guide the management of unhealed wounds/PPS in patients with IBD. This evidence could be used to inform new guidelines for the treatment of unhealed wounds/PPS following proctectomy in IBD, which are currently lacking. The TOpClass consortium is building the foundations of these studies through development of the standardised understanding and definitions required.

In addition to the limitations described previously, most clinical studies investigating treatments for unhealed wounds/PPS were published prior to the widespread use of advanced therapies in IBD. As a result, the outcomes of many treatments reported in these studies may not be generalisable to modern cohorts of patients with IBD. Although there is some evidence from a recent meta-analysis suggesting that the use of biologics does not have an effect on the rate of wound healing after proctectomy [4], the role of biologics as a treatment for unhealed wounds/PPS alone or as an adjunct to surgical treatments (and perhaps in particular classes of patients) requires further investigation.

The measurement of endpoints throughout these case series was heterogenous. Definitions of what constituted healing were varied, sometimes reported as a binary “healed/unhealed”, and sometimes with further gradations. Of note, one study included a patient with an unwillingness to go ahead with additional treatment as a “poor result” [28]. Under the TOpClass classification this may indicate a realignment of patient goals, rather than a failure of treatment. In several studies after the intervention was performed, it was determined that the patient had an enteroperineal fistula contrary to previous imaging findings—this could be considered a complication of the closure attempt, or a previously unseen but persistent finding [36]. Additionally, multiple treatments were often required to achieve what authors consider to be a satisfactory outcome. In this patient cohort, it was not unusual for patients to have 5–35 procedures prior to the intervention under study [37]. Duration of follow-up was often less than 12 months and varied significantly across participants in a single series.

Only one case series, Lansdorp et al., used any PROMs to assess the efficacy of treatment, in a limited series of three patients undergoing HBOT. Of these patients, one had a clinically persistent sinus following treatment but had marked improvement across a panel of generic PROMs.

### Classification systems

Perhaps the most established classification system for unhealed wounds/PPS is the Watts-Goligher classification, which was originally described in 1966 as part of a retrospective case series assessing complications following pan-proctocolectomy for UC. They defined any perineal wound that was not healed for longer than 6 months a PPS. Although useful, this does not cover the variety and complexity of wound healing issues that may occur and thus does not aid clinicians in selecting the most appropriate treatment option. More recently, for the purpose of a retrospective cohort study assessing PPS after proctocolectomy for CD Yamamoto expanded on this classification into three groups—‘early healing’ within 12 weeks of proctocolectomy, ‘delayed healing’ within 12 weeks and 6 months of proctocolectomy, and persistent sinus if the wound remains unhealed for more than 6 months. Anthony et al. defined chronic wounds as those persisting for longer than 1 month [14].

**Fig. 3:**
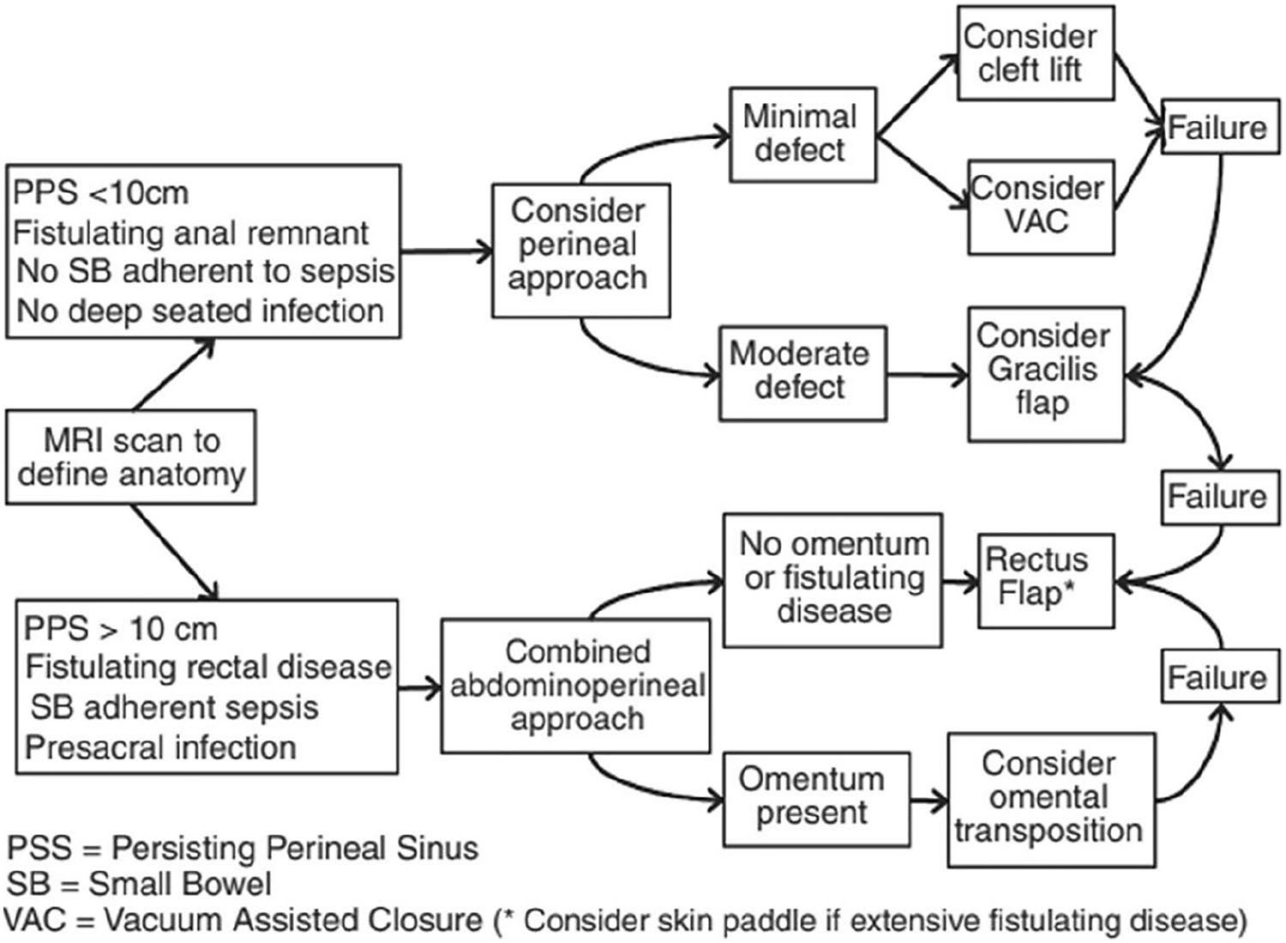
Management strategy from Wilson et al. (reproduced with permission)

Wilson et al. present a classification system incorporated into a treatment strategy for the management of chronic pelvis sepsis following proctectomy (Fig. 3) [35]. On the basis of an initial MRI, this categorises PPS into two groups, superficial (< 10 cm) without presacral sepsis and adherent small bowel, and > 10 cm with presacral infection and adherent small bowel. These groups are recommended for different treatment options, with the former more suitable for local surgical options such as cleft lift, and the latter for larger options such as omentoplasty or RAM flaps. There is some evidence from another retrospective study to indicate that short (< 10 cm) sinuses are more likely to resolve with surgical treatment than longer sinuses [33].

The TOpClass classification relates specifically to pCD. In this classification system, perineal symptoms after proctectomy are described as Class 4 disease, which is subdivided into Class 4a and Class 4b. Class 4a disease includes symptomatic unhealed wounds/PPS that can be treated with a combination of medical and surgical interventions, with the primary goal being complete sinus closure. In contrast, Class 4b refers to patients experiencing persistent symptoms from sinuses or wounds that impact their quality of life but are not suitable for surgical repair, or when the primary objective is symptom management rather than closure. This classification system takes a novel approach in comparison with other classification systems for unhealed wounds/PPS, incorporating patient goals, which allows for a more holistic process aligned with shared clinical decision-making. At a recent consortium meeting, the need for a more detailed classification of Class 4 disease was confirmed and this work is underway.

Studies have used various terms for the unhealed wounds/PPS that they describe. Some terms, such as delayed healing, imply a time-dependent problem and an ultimate resolution. Others, such as poorly healing, imply a mechanism of action preventing the wound from healing. Sinus implies a contained ‘tunnel’ which may also be misleading and of course these features of unhealed wound/PPS overlap and appear to different degrees. For the purposes of future research it will be important to 1. determine a universal nomenclature, 2. identify difference in the pathogenesis and/or treatment options and outcomes for different unhealed wounds and sinuses, and 3. utilise a sufficiently precise classification to determine those differences. All three components of this work are underway within the TOpClass consortium, as well as other elements necessary to inform an interventional trial in this group of patients; but in the context of this review, the mixed naming and descriptions add doubt.

### Limitations of the systematic review process

This systematic review was carried out in accordance with PRISMA reporting. A possible limitation of this systematic review is that it was limited to full text articles only, as it was considered unlikely an abstract would contain the requisite methodological detail and results data to answer the research question.

### Summary: Implications of the results for practice, policy and research

There are multiple medical and surgical treatment options available for the management of unhealed wounds/PPS post-proctectomy. However, the studies supporting these treatments are generally retrospective with poor methodological quality, heterogenous measurement of key endpoints, and limited usage of PROMs. A more detailed classification system, perhaps based on TOpClass, may help to assist in patient selection for treatment in both a clinical and research setting. The development of clearly defined patient-derived endpoints and objective outcome measurements will facilitate meaningful prospective studies assessing symptom control, surgical repair and combined medical and surgical interventions and algorithms to improve outcomes for these patients.

## Data Availability

No datasets were generated or analysed during the current study.

## Declarations

### AI

During the preparation of this work, the author used PaperPal to proofread the grammar and punctuation. After using this tool/service, the author(s) reviewed and edited the content as needed and took full responsibility for the publication’s content.
